# Supplementary material for: Asynchronous magnetic resonance elastography: Shear wave speed reconstruction using noise correlation of incoherent waves
Source: Magn Reson Med. 2022 Oct 27;89(3):990–1001. doi: 10.1002/mrm.29502 (PMC9792433; doi:10.1002/mrm.29502)
Supplement: Supplementary file 1 — DATA S1 MATLAB code used to generate the numerical simulations of Figure 1 [file MRM-89-990-s002.zip › k-Wave/helpfiles/kspaceFirstOrderAS.html]

kspaceFirstOrderAS :: Functions (k-Wave)


# kspaceFirstOrderAS

Axisymmetric time-domain simulation of wave propagation.

## Syntax

```
sensor_data = kspaceFirstOrderAS(kgrid, medium, source, sensor)
sensor_data = kspaceFirstOrderAS(kgrid, medium, source, sensor, ...)
```

## Description

`kspaceFirstOrderAS` simulates the time-domain propagation of compressional waves through an axisymmetric homogeneous or heterogeneous acoustic medium. The code is functionally very similar to `kspaceFirstOrder2D`. However, a 2D axisymmetric coordinate system is used instead of a 2D Cartesian coordinate system. In this case, `x` corresponds to the axial dimension, and `y` corresponds to the radial dimension as shown below. In the radial dimension, the first grid point corresponds to the grid origin, i.e., `y = 0`. In comparison, for `kspaceFirstOrder2D`, the Cartesian point `y = 0` is in the middle of the computational grid.

The input structures `kgrid`, `medium`, `source`, and `sensor` are defined in exactly the same way as for `kspaceFirstOrder2D`. However, computationally, there are several key differences. First, the axisymmetric code solves the coupled first-order equations accounting for viscous absorption (not power law), so only `medium.alpha_power = 2` is supported. This value is set by default, and doesn't need to be defined. This also means that `medium.alpha_mode` and `medium.alpha_filter` are not supported. Second, for a homogeneous medium, the k-space correction used to counteract the numerical dispersion introduced by the finite-difference time step is not exact (as it is for the other fluid codes). However, the approximate k-space correction still works very effectively, so dispersion errors should still be small. See `kspaceFirstOrder2D` for additional details on the function inputs.

In the x-dimension (axial), the FFT is used to compute spatial gradients. In the y-dimension (radial), two choices of symmetry are possible. These are whole-sample-symmetric on the interior radial boundary (y = 0) and either whole-sample-symmetric or whole-sample-asymmetric on the exterior radial boundary. These are abbreviated WSWA and WSWS. The WSWA and WSWS symmetries are implemented using both discrete trigonometric transforms (DTTs), and via the FFT by manually mirroring the domain. The latter options are abbreviated as WSWA-FFT and WSWS-FFT. The WSWA/WSWS options and the corresponding WSWA-FFT/WSWS-FFT options agree to machine precision. When using the PML, the choice of symmetry doesn't matter, and all options give very similar results (to several decimal places). Computationally, the DTT implementations are more efficient, but require additional compiled MATLAB functions (not currently part of k-Wave). The symmetry can be set by using the optional input `'RadialSymmetry'`. The WSWA-FFT symmetry is set by default.

## Inputs

The minimum fields that must be assigned to run an initial value problem (for example, a photoacoustic forward simulation) are marked with a \*.

|  |  |
| --- | --- |
| `kgrid*` | k-Wave grid object returned by `kWaveGrid` containing axisymmetric and k-space grid fields |
| `kgrid.t_array*` | evenly spaced array of time values [s] (set to `'auto'` by `kWaveGrid`) |
|  |  |
| `medium.sound_speed*` | sound speed distribution within the acoustic medium [m/s] |
| `medium.sound_speed_ref` | reference sound speed used within the k-space operator (phase correction term) [m/s] |
| `medium.density*` | density distribution within the acoustic medium [kg/m^3] |
| `medium.BonA` | parameter of nonlinearity |
| `medium.alpha_coeff` | power law absorption coefficient [dB/(MHz^y cm)] |
| `medium.alpha_sign` | scalar used to control the sign of the absorption term in the equation of state |
|  |  |
| `source.p0*` | initial pressure within the acoustic medium |
| `source.p` | time varying pressure at each of the source positions given by `source.p_mask` |
| `source.p_mask` | binary matrix specifying the positions of the time varying pressure source distribution |
| `source.p_mode` | optional input to control whether the input pressure is injected as a mass source or enforced as a dirichlet boundary condition; valid inputs are `'additive'` (the default) or `'dirichlet'` |
| `source.ux` | time varying particle velocity in the x-direction (axial) at each of the source positions given by `source.u_mask` |
| `source.uy` | time varying particle velocity in the y-direction (radial) at each of the source positions given by `source.u_mask` |
| `source.u_mask` | binary matrix specifying the positions of the time varying particle velocity distribution |
| `source.u_mode` | optional input to control whether the input velocity is applied as a force source or enforced as a dirichlet boundary condition; valid inputs are `'additive'` (the default) or `'dirichlet'` |
|  |  |
| `sensor.mask*` | binary matrix or a set of Cartesian points where the pressure is recorded at each time-step |
| `sensor.record` | cell array of the acoustic parameters to record in the form `sensor.record = {'p', 'u', ...}`; valid inputs are:    `'p'` (acoustic pressure)    `'p_max'` (maximum pressure)    `'p_min'` (minimum pressure)    `'p_rms'` (RMS pressure)    `'p_final'` (final pressure field at all grid points)    `'p_max_all'` (maximum pressure at all grid points)    `'p_min_all'` (minimum pressure at all grid points)    `'u'` (particle velocity)    `'u_max'` (maximum particle velocity)    `'u_min'` (minimum particle velocity)    `'u_rms'` (RMS particle velocity)    `'u_final'` (final particle velocity field at all grid points)    `'u_max_all'` (maximum particle velocity at all grid points)    `'u_min_all'` (minimum particle velocity at all grid points)    `'u_non_staggered'` (particle velocity on non-staggered grid)    `'I'` (time varying acoustic intensity)    `'I_avg'` (average acoustic intensity) |
| `sensor.record_start_index` | time index at which the sensor should start recording the data specified by `sensor.record` (default = 1) |
| `sensor.time_reversal_boundary_data` | time varying pressure enforced as a Dirichlet boundary condition over `sensor.mask` |
| `sensor.frequency_response` | two element array specifying the center frequency and percentage bandwidth of a frequency domain Gaussian filter applied to the `sensor_data` |

**Note**  For a heterogeneous medium, `medium.sound_speed` and `medium.density` must be given in matrix form with the same dimensions as `kgrid`. For a homogeneous medium, these can be given as scalar values. If the medium is homogeneous and velocity inputs or outputs are not required, it is not necessary to specify `medium.density`.

## Optional Inputs

Optional 'string', value pairs that may be used to modify the default computational settings.

| Input | Valid Settings | Default | Description |
| --- | --- | --- | --- |
| `'CartInterp'` | `'linear'` `'nearest'` | `'linear'` | Interpolation mode used to extract the pressure when a Cartesian sensor mask is given. If set to `'nearest'` and more than one Cartesian point maps to the same grid point, duplicated data points are discarded and `sensor_data` will be returned with less points than that specified by `sensor.mask`. |
| `'CreateLog'` | *(Boolean scalar)* | `false` | Boolean controlling whether the command line output is saved using the diary function with a date and time stamped filename. |
| `'DataCast'` | *(string of data type)* | `'off'` | String input of the data type that variables are cast to before computation. For example, setting to `'single'` will speed up the computation time (due to the improved efficiency of `fftn` and `ifftn` for this data type) at the expense of a loss in precision. This variable is also useful for utilising GPU parallelisation through libraries such as the Parallel Computing Toolbox by setting `'DataCast'` to `'gpuArray-single'`. |
| `'DataRecast'` | *(Boolean scalar)* | `false` | Boolean controlling whether the output data is cast back to double precision. If set to false, `sensor_data` will be returned in the data format set using the `'DataCast'` option. |
| `'DisplayMask'` | *(binary matrix)* or | `sensor.mask` | Binary matrix overlaid onto the animated simulation display. Elements set to 1 within the display mask are set to black within the display. |
| `'HDFCompressionLevel'` | *(Integer scalar)* | `0` | Compression level used for writing the input HDF5 file when using `'SaveToDisk'` or `kspaceFirstOrderASC`. Can be set to an integer between 0 (no compression) and 9 (maximum compression). The compression is lossless. Increasing the compression level will reduce the file size if there are portions of the medium that are homogeneous, but will also increase the time to create the HDF5 file. |
| `'LogScale'` | *(Boolean scalar)* or *(numeric scalar)* | `false` | Boolean controlling whether the pressure field is log compressed before display. The data is compressed by scaling both the positive and negative values between 0 and 1 (truncating the data to the given plot scale), adding a scalar value (compression factor) and then using the corresponding portion of a log10 plot for the compression (the negative parts are remapped to be negative thus the default color scale will appear unchanged). The amount of compression can be controlled by adjusting the compression factor which can be given in place of the Boolean input. The closer the compression factor is to zero, the steeper the corresponding part of the log10 plot used, and the greater the compression (the default compression factor is 0.02). |
| `'MeshPlot'` | *(Boolean scalar)* | `false` | Boolean controlling whether `mesh` is used in place of `imagesc` to plot the pressure field. When `'MeshPlot'` is set to `true`, the default display mask is set to `'off'`. |
| `'MovieArgs'` | *(string cell array)* | `{}` | Settings for `VideoWriter`. Parameters must be given as {'param', value, ...} pairs within a cell array (default = {}), where 'param' corresponds to a writable property of a VideoWriter object. |
| `'MovieName'` | *(string)* | `'date-time-kspaceFirstOrder2D'` | Name of the movie produced when `'RecordMovie'` is set to `true`. |
| `'MovieProfile'` | *(string)* | `'Uncompressed AVI'` | Profile input passed to `VideoWriter`. |
| `'PlotFreq'` | *(integer numeric scalar)* | `10` | The number of iterations which must pass before the simulation plot is updated. |
| `'PlotLayout'` | *(Boolean scalar)* | `false` | Boolean controlling whether a four panel plot of the initial simulation layout is produced (initial pressure, sensor mask, sound speed, density). |
| `'PlotPML'` | *(Boolean scalar)* | `true` | Boolean controlling whether the perfectly matched layer is shown in the simulation plots. If set to `false`, the PML is not displayed. |
| `'PlotScale'` | *(numeric two element vector)* or `'auto'` | `[-1, 1]` | [min, max] values used to control the scaling for `imagesc` (visualisation). If set to `'auto'`, a symmetric plot scale is chosen automatically for each plot frame. |
| `'PlotSim'` | *(Boolean scalar)* | `true` | Boolean controlling whether the simulation iterations are progressively plotted. |
| `'PMLAlpha'` | *(numeric scalar or three element vector)* | `2` | Absorption within the perfectly matched layer in Nepers per grid point. |
| `'PMLInside'` | *(Boolean scalar)* | `true` | Boolean controlling whether the perfectly matched layer is inside or outside the grid. If set to `false`, the input grids are enlarged by `PMLSize` before running the simulation. |
| `'PMLSize'` | *(integer numeric scalar or three element vector)* | `10` | Size of the perfectly matched layer in grid points. By default, the PML is added evenly to all sides of the grid, however, both `PMLSize` and `PMLAlpha` can be given as two element arrays to specify the x and y properties, respectively. To remove the PML, set the appropriate `PMLAlpha` to zero rather than forcing the PML to be of zero size. |
| `'RadialSymmetry'` | *(string)* | `'WSWA-FFT'` | Radial symmetry assumed at the inner and outer domain boundaries by the simulation, where W: whole sample, H: half sample, S: symmetric, A: antisymmetric. Valid inputs are `'WSWA-FFT'`, `'WSWS-FFT'`, `'WSWA'`, and `'WSWS'`. The first two options are computed using the FFT by mirroring the domain appropriately. The final two options are computed using the discrete cosine and sine transformation and require additional functions (not currently part of k-Wave). |
| `'RecordMovie'` | *(Boolean scalar)* | `false` | Boolean controlling whether the displayed image frames are captured and stored as a movie using `VideoWriter`. |
| `'Smooth'` | *(Boolean scalar or three element vector)* | `[true, false, false]` | Boolean controlling whether `source.p0`, `medium.sound_speed`, and `medium.density` are smoothed using `smooth` before computation. `'Smooth'` can either be given as a single Boolean value or as a 3 element array to control the smoothing of `source.p0`, `medium.sound_speed`, and `medium.density`, independently. |

## Outputs

If `sensor.record` is not defined by the user:

|  |  |
| --- | --- |
| `sensor_data` | time varying pressure recorded at the sensor positions given by `sensor.mask` |

If `sensor.record` is defined by the user:

|  |  |
| --- | --- |
| `sensor_data.p` | time varying pressure recorded at the sensor positions given by `sensor.mask` (returned if `'p'` is set) |
| `sensor_data.p_max` | maximum pressure recorded at the sensor positions given by `sensor.mask` (returned if `'p_max'` is set) |
| `sensor_data.p_min` | minimum pressure recorded at the sensor positions given by `sensor.mask` (returned if `'p_min'` is set) |
| `sensor_data.p_rms` | rms of the time varying pressure recorded at the sensor positions given by `sensor.mask` (returned if `'p_rms'` is set) |
| `sensor_data.p_final` | final pressure field at all grid points within the domain (returned if `'p_final'` is set) |
| `sensor_data.p_max_all` | maximum pressure recorded at all grid points within the domain (returned if `'p_max_all'` is set) |
| `sensor_data.p_min_all` | minimum pressure recorded at all grid points within the domain (returned if `'p_min_all'` is set) |
| `sensor_data.ux` | time varying particle velocity in the x-direction recorded at the sensor positions given by `sensor.mask` (returned if `'u'` is set) |
| `sensor_data.uy` | time varying particle velocity in the y-direction recorded at the sensor positions given by `sensor.mask` (returned if `'u'` is set) |
| `sensor_data.ux_max` | maximum particle velocity in the x-direction recorded at the sensor positions given by `sensor.mask` (returned if `'u_max'` is set) |
| `sensor_data.uy_max` | maximum particle velocity in the y-direction recorded at the sensor positions given by `sensor.mask` (returned if `'u_max'` is set) |
| `sensor_data.ux_min` | minimum particle velocity in the x-direction recorded at the sensor positions given by `sensor.mask` (returned if `'u_min'` is set) |
| `sensor_data.uy_min` | minimum particle velocity in the y-direction recorded at the sensor positions given by `sensor.mask` (returned if `'u_min'` is set) |
| `sensor_data.ux_rms` | rms of the time varying particle velocity in the x-direction recorded at the sensor positions given by `sensor.mask` (returned if `'u_rms'` is set) |
| `sensor_data.uy_rms` | rms of the time varying particle velocity in the y-direction recorded at the sensor positions given by `sensor.mask` (returned if `'u_rms'` is set) |
| `sensor_data.ux_final` | final particle velocity field in the x-direction at all grid points within the domain (returned if `'u_final'` is set) |
| `sensor_data.uy_final` | final particle velocity field in the y-direction at all grid points within the domain (returned if `'u_final'` is set) |
| `sensor_data.ux_max_all` | maximum particle velocity in the x-direction recorded at all grid points within the domain (returned if `'u_max_all'` is set) |
| `sensor_data.uy_max_all` | maximum particle velocity in the y-direction recorded at all grid points within the domain (returned if `'u_max_all'` is set) |
| `sensor_data.ux_min_all` | minimum particle velocity in the x-direction recorded at all grid points within the domain (returned if `'u_min_all'` is set) |
| `sensor_data.uy_min_all` | minimum particle velocity in the y-direction recorded at all grid points within the domain (returned if `'u_min_all'` is set) |
| `sensor_data.ux_non_staggered` | time varying particle velocity in the x-direction recorded at the sensor positions given by `sensor.mask` after shifting to the non-staggered grid (returned if `'u_non_staggered'` is set) |
| `sensor_data.uy_non_staggered` | time varying particle velocity in the y-direction recorded at the sensor positions given by `sensor.mask` after shifting to the non-staggered grid (returned if `'u_non_staggered'` is set) |
| `sensor_data.Ix` | time varying acoustic intensity in the x-direction recorded at the sensor positions given by `sensor.mask` (returned if `'I'` is set) |
| `sensor_data.Iy` | time varying acoustic intensity in the y-direction recorded at the sensor positions given by `sensor.mask` (returned if `'I'` is set) |
| `sensor_data.Ix_avg` | average acoustic intensity in the x-direction recorded at the sensor positions given by `sensor.mask` (returned if `'I_avg'` is set) |
| `sensor_data.Iy_avg` | average acoustic intensity in the z-direction recorded at the sensor positions given by `sensor.mask` (returned if `'I_avg'` is set) |

## Examples

- Simulations In An Axisymmetric Coordinate System

## See Also

`kspaceFirstOrder1D`, `kspaceFirstOrder2D`, `kspaceFirstOrder3D`, `kWaveGrid`
